# Navigating new organisation forms: a qualitative study of primary care networks

**DOI:** 10.3399/BJGPO.2021.0092

**Published:** 2022-03-09

**Authors:** Maria Kordowicz, Becky Malby, Kieran Mervyn

**Affiliations:** 1 Community and Health Research Unit, University of Lincoln, Lincoln, UK; 2 London South Bank University, Health Systems Innovation Lab, London, UK

**Keywords:** primary care networks, primary health care, general practice, collaboration, health systems, health organisation, general practitioners

## Abstract

**Background:**

NHS England (NHSE) instigated primary care networks (PCNs) as a collaboration of general practices working together at scale to improve population health in the local community.

**Aim:**

To capture GP PCN leaders’ perceptions of the opportunities and pitfalls of PCNs, as well as points of learning, during their inception and development, in order to guide the future development of PCN form and function.

**Design & setting:**

A qualitative study in UK primary care.

**Method:**

Nine PCN GP leaders were interviewed in depth to gather their views and experiences of PCNs. In addition, 31 free-text survey responses pertaining to how participants perceived the purpose of PCNs were collated.

**Results:**

Four key themes were identified: defining purpose and managing ambiguity; bureaucracy versus local autonomy; relational working; and facilitative leadership. The need for purpose setting to remain adaptive was seen as crucial in avoiding the constraints of too rigid a structure in order to retain local ownership, while remaining focused around meeting complex population needs and reducing variation. Participants reported navigating their way through striking a balance between the ‘top-down’ mandate and recognising local need. Of importance to the success of PCNs was the necessity of effective relational working and facilitative leadership.

**Conclusion:**

While the desire to be proactive and collaborative was emphasised by the PCN leaders, the importance of distributed leadership and time given to building trust and effective working relationships within new organisational forms cannot be underestimated.

## How this fits in

As PCNs are newly created, there is a gap in knowledge concerning how newly appointed PCN leaders navigate these new organisational forms. This study captures insights regarding how PCNs have been experienced by PCN leaders, their perceived limitations and opportunities, as well as recommendations for future PCN and PCN leader development.

## Introduction

In 2019, NHSE instigated PCNs as a collaboration of general practices working together at scale with populations ranging from 30 000–50 000 to improve their health. No doubt in light of pandemic pressures, the need for collaboration has become even more crucial. The aim of PCNs is to create more sustainable holistic primary health care at the population level to better meet challenges of complexity and promote best use of resource within an integrated care system (ICS).^1^

Although GPs have worked in a range of collaborative formations over time (for example, GP fundholding, federations, and clusters), PCNs are an attempt by NHSE to formalise collaboration without creating new statutory organisations. There is a body of literature highlighting the benefits of collaborations in general practice, and between GPs and other healthcare professionals formed organically over several years built on mutual trust, rather than ones imposed ‘top-down’ without a shared clear purpose.^2–5^ PCNs are part of a wider transition in the NHS, which in the UK is following the global healthcare system trend of a mixed model of organisation in an increasingly interdependent system, comprising competition for procurement, strong regulation for baseline performance, and collaboration through networks to address complex needs.^6^

In terms of the emerging academic literature concerning PCNs in England, an early study of GPs working in networks highlighted tensions between the prescribed structure and GPs’ scope to implement local solutions, while also capturing optimism about their potential to improve care quality.^7^ Owing to the recency of the PCN initiative, there is a paucity of literature on the experiences of those at the forefront of implementation in navigating the new PCN organisational forms, and the mixed messaging of self-organisation and determination versus prescribed services and direction. Meanwhile, published opinion pieces tend to highlight ‘mounting unrest’ and clinician uncertainty around the initiative,^8,9^ with arguably more measured responses from professional bodies and think tanks recognising the potential benefits of embedding more person-centred care and helping to tackle health inequalities.^1^ The need to avoid ‘top-down’ imposition of a given structure so as not to undermine the potential for local targeted solutions has also been emphasised.^10^

There is a small comparative body of literature typically attempting to quantify the clinical effectiveness of PCNs. For instance, in helping to improve population diabetes management through shared resource in Singapore^11^ and, in a similar vein, a primary care collaborative model was associated with lower risks of hospital admission or emergency department visits for diabetes-specific presentations in Canada.^12^ Notably, the need for general practice networks to be granted autonomy and agency is seen as key in helping them tackle complex healthcare challenges in Australia.^13^

Lastly, the broad business and management literature surrounding the effectiveness, or otherwise, of collaboration can also be drawn on, particularly when mandated within a brief space of time as in the instance of PCNs. It is argued that trust, which is key to effective collaborative working, evolves over time within strategic alliances, and is the product of complex individual and team-level interactions.^14^ Moreover, organisational relationship histories have been cited as an explanatory factor for explaining the effectiveness of inter-organisational working, for instance, in procurement and purchasing.^15^ It follows that the length of the relationship plays a part, whereby the longer the organisations (in this case general practices) have worked together, behavioural uncertainty is less likely and relationship quality is improved. The arguably short timeframes for the formation of PCNs raised questions about the extent to which effective collaborative working could be achieved in this time period, as well as the purpose and impact of that collaboration, and it was hoped to gain an insight into this through this study. In addition, the study sought to find out how PCNs were interpreting their purpose in order to develop their organisational form.

It is of interest to understand the extent to which these pre-existing understandings of navigating and developing new collaborative entities apply in the context of PCNs. Indeed, it is argued that successful collaborations are determined by a variety of factors inherent in the broader social structures, including a clear shared purpose and narrative, the focus on distributed leadership, data-enabled adaptation, and shared learning and development.^16^ Hence, the usefulness of further in-depth qualitative studies that can mine the richness of these factors. This study therefore sought to address a knowledge gap by exploring the perceptions and experiences of GP leads of the PCN initiative and how its implementation has worked in practice.

This study set out to explore the views of GPs whose general practices were at the forefront of establishing PCNs in the NHS. The aim of this study was to gauge what barriers and opportunities the senior clinician participants were encountering in this process, along with gaining an insight into how the new PCN organisational forms were being experienced and navigated.

## Method

To address the aims, this study sought to explore PCN leaders’ perceptions of the new PCN organisational forms and of navigating their roles within them through in-depth qualitative interviews. Using in-depth, semi-structured interviews was considered the most appropriate method for examining professionals’ perspectives and views about PCNs. Interviews have been used previously to explore the perspectives of healthcare professionals and have been proven to be an effective data-collection method.^17,18^ All methods were performed in accordance with London South Bank University institutional ethics guidelines and regulations.

Nine participants were recruited to be interviewed at opportunity. The participants were approached through network contacts and consisted of nine senior clinicians whose general practices were at the forefront of PCN implementation at the time of the study. Participants were provided with an information sheet and consent was sought for participation in the interviews and survey. The interviews, which were conducted face-to-face, audiorecorded, and transcribed, were a mean average 80 minutes in length. The semi-structured and open-ended interview questions explored participants’ understanding of the purpose of their PCNs; gauged how well they saw PCNs as fulfilling that purpose; and illuminated any barriers and opportunities they had encountered in establishing PCNs.

In addition, an online free-text survey was sent to PCN leaders, which attracted 31 responses. The aim of the survey was to build a sense of how responders defined the purpose of their PCNs by asking them to respond using free text to the following:

- please provide your PCN’s agreed purpose;
- what is the focus of your PCNs work this year? Where are you putting your energy with the time you have? And
- what is the focus for your development funding this year? What have you chosen to spend this funding on?

The participant interview transcript and survey response data were coded blind, utilising codes in NVivo (version 10). During the process of analysis, the guidelines developed by Braun and Clarke^19^ were applied.

Following the repeated reading of transcripts and survey data, key issues and themes emerging from the data were identified, shaped, and developed further through iterative team discussion. This stage allowed for the exploration of areas of inter-rater agreement, as well as for the definition of overarching themes. The analysis took on an inductive and deductive hybrid,^20^ whereby the authors‘ prior knowledge of health systems shaped the theme interpretation and discussion, yet their definition was led by the synthesis of the participant responses.

## Results

Overarching themes are presented with illustrative quotes from both the interviews and free-text survey responses.

### Theme 1: Defining purpose and managing ambiguity

The participants’ PCNs were at varying stages of development or organisational maturity.

For some, the purpose was still evolving:

*‘* *I think that’s still emerging, I think for me though, it’s about how we work at scale to maximise efficiencies and how we start to provide place-based care.* *’* (Participant [Pp]8)

This was navigated by some participants with a tendency to express the purpose of PCNs and their own PCNs in a more exploratory manner, adding their own ’agenda’ or interpretation to the definition of purpose. However, the need for the purpose to remain adaptive was seen as crucial in avoiding the constraints of too rigid a structure:

*‘* *I’m trying to encourage the potential to avoid the soft side of PCNs, particularly where money is involved, people tend to focus much more on the structure and people tend to pretend they know what a PCN is meant to be or where we are meant to go, but the risk is that they create so much structure that it’s no longer fit for purpose* *.* *’* (Pp4)

Purpose-setting appeared to be something that was exploratory and self-defined for those participants, as a response to ambiguities around the aims set for PCNs nationally:

*‘* *It’s very much driven by what’s going on nationally, but it seems to have drifted and they have left it to the PCNs to decide* *.* *’* (Pp5)

*‘* *I don’t think we’ve finally answered this question* [of what our purpose is] *in our network yet* *.* *’* (Pp13, survey)

However, it was identified that without a clear purpose PCNs would become reactive rather than proactive, and this was a pitfall PCN leaders wanted to avoid:

*‘There needs to be proactive and preventative care and in terms of managing our workload so the right patients are seen by the right people.* *’* (Pp5)

*‘By that I mean we look at the pop we serve, identify what the problems are and come up with solutions for the problems that our pop has. So we need to move from a reactive to a proactive model of care.* *’* (Pp2)

Other participants stated their PCN’s purpose with confidence and clarity:

*‘We have two* [aims]*, one is to address the health needs of our community, and two is to provide an attractive place of work for primary care staff* *.* *’* (Pp1)

*‘* *Better access to more health services and more sustainable general practices* *.* *’* (Pp6, survey)

The commonalities across the stated purpose by the majority of participants referred to recognising and addressing the health needs of the local community through collaboration, as well as improving the quality of care at a population level, for instance:

*‘The core purpose of the networks is to get practices to collaborate so that we can deliver population health management to the population we serve* *.* *’* (Pp2)

However, a range of aims was stated, including sustainability, a family-based approach, democratic employee relations, and improving GP working lives:

*‘* *...* *the top-line vision is to deliver a quality service … combining our service that they wrap around the patient … improving work/life balance for GPs and the community* *.* *’* (Pp3)

Working at scale was perceived as an opportunity to meet complex needs through integration and reinvestment, including by improving access for marginalised people:

*‘* *...* *to reinvest funds in community and staff to improve health, help marginalised groups.* *’* (Pp7, survey)

Overall, the majority of participants appeared to have embraced the NHSE mandate through espousing the policy purpose of PCNs:

*‘* *The purpose is to bring GPs back to working together to provide care which they can have some efficiencies of scale.* *’* (Pp6)

### Theme 2: Bureaucracy versus local autonomy

This theme builds on the tensions evident in securing a clear purpose, and refers to the participants seemingly navigating their way through striking a balance between the ‘top-down’ mandate and recognising local need. This appeared to be an emergent and ongoing process:

*‘* *...* *on a national level, there is this kind of global rigidity with global flexibility being talked about but then when I go to my local area, they are not empowered to be flexible* *…* *rigidity doesn’t work in that context we have to be flexible.* *‘* (Pp8)

There were clear tensions between the purposes that related to structures, delivery, and contracting (primarily set ‘top-down’); and those that related to meeting needs (primarily the interest of the PCNs themselves). Concerns were expressed that the dominance of funding and central attention on new roles and structures would lead to a traditional delivery organisation rather than a flexible and adaptive new model of care to meet complex needs that require collaboration:

*‘The problem is that lots of people see PCNs as just a name and the risk is that they ignore the network part of the name in a rush to get a structure and tendency to say* *“* *just tell us what we need to do and when we need to do it* *”* *, rather than just let people discover what they need to do and allow them to change things as they go along.* *’* (Pp17, survey)

*‘* *…* *as* *primary care networks* *mature their level of autonomy and control should increase, and their freedom to act should increase* *…* *the NHS is very top down* *—* *the NHS is very command and control.* *’* (Pp21, survey)

Further, the participants conveyed their responses to some of the more transactional demands and highlighted the need to establish their own power base within the wider institutional context:

*‘* *...* *we believe that the integrated care system should have specific clinical representation so that it doesn’t just become it determining to the PCNs what is needed but more that the PCNs are telling the integrated care system.* *’* (Pp5)

The potential for contractual obligations to be stifling was raised, along with the need to be enterprising beyond formal structures in order to make a change. Responses of participants conveyed the differences between emergent PCN-determined purpose and the implementation of PCNs through clinical commissioning groups (CCGs), which used a contracting and performance management relationship with primary care:

*‘Contracts are absolutely pointless. Contracts are often used by people to prevent progress and change and as soon as someone says that’s not in my contract, you know you’re onto a loser and know you need to go right back to trying to build the shared purpose and common goal. And most of what we’ve managed to achieve is sat outside of contracts where we’ve just ignored the contracts and with permission and just said let’s get on with that.* *’* (Pp7)

Further, some of the participants presented themselves as having to navigate between the transactional and the purposeful:

*‘But now actually we are in a position where we are going to have to sub-contract to every single member practice because they are the ones with the DES* [Directed Enhanced Services]*. So then you get into implications around things like that, and NHS pensions* *…* *that’s the chunky stuff that I think is a threat, because a GP is just going to go* *—* *do you know what, I just can’t, I ain’t got time* *…* *and you lose the ownership.* *’* (Pp9)

However, within this expression of the weight of bureaucracy, it was recognised that lines of accountability needed to be clearer, particularly for leaders attempting to stimulate innovation:

*‘* *...* *they’ve literally been around more leadership as opposed to you know the functions of accountability and the balancing of your regulatory sort of role against your innovation. There’s none of that really, just that is what helps them.* *’* (Pp9)

Further, it was felt that in order to achieve local autonomy, access to informatics for the purpose of quality improvement at the local level was key so as to do away with the bureaucracy often surrounding data access negotiations:

*‘* *…* *I want to see each primary care network have its access to its own data analyst and QI* [quality improvement] *team* *.* *’* (Pp7)

### Theme 3: Relational working

Given that collaborative working is the explicit premise of PCNs, it is perhaps unsurprising that overwhelmingly the participants recognised the importance of relationships in implementing and developing them. Relational working was seen as both a challenge and an opportunity:

*‘* *...* *the challenges, are firstly, relational and where there are good relationships* *…* *primary care networks* *will clearly flourish and develop quickly* *.* *’* (Pp6)

Developing trust and effective decision making was perceived as underpinning successful working relationships, in order to achieve engagement with the PCN initiative and its local projects:

*‘* *...* *the biggest challenge is going to be relationships and trust. Until trust is developed then people won’t engage* *…* *If you put ten people in a room and call them a network, and now just go and function* *—* *that is not going to happen. Time, breathing space* *,* *and head space to help people to get together and know each other.* *’* (Pp5)

*‘* *It was a bit touch and go in places but now we’ve got that trusty relationship* *—* *we feel part of a bigger team, so it’s really good.* *’* (Pp9)

In addition, at the core of forming effective relational working, was challenging siloed thinking and behaviours:

*‘* *...* *it’s about relationship building; it would be that the political and contractual environment has kind of pushed us into working in silos* *…* *I think we need to put work and therefore investment into building relationships* *…* *But if present relationships aren’t in place, you drop the plan you’ve actually got to work on building the relationship before you can deliver it, so it just slows things down.* *’* (Pp8)

PCN leaders were recognised as the enablers of positive relational working:

*‘* *...* *if we have leaders whose style is to build relationships, then they are very well prepared* *.* *’* (Pp1)

### Theme 4: Facilitative leadership

Beyond improving working relationships within the PCN collaboration, participants talked of the style of leadership required for this new organisational form to be successful. The participants saw this being achieved by breaking away from a transactional style of leadership, which had no place in the new landscape:

*‘* *...* *who are the leaders that are going to facilitate this transformative process?* *…* *some of those don’t currently recognise themselves as leaders* *—* *some do, but have got very minimal leadership experience: some people think themselves a leader and call themselves a leader and get paid as a leader and recognised as a leader and they are not.* *’* (Pp1)

*‘PCN is something not hierarchical, more inclusive and more collaborative and giving space to build and try new innovative ideas. The leader probably doesn’t have a command and control approach, but more of a role of helping to build other people and being more like a conductor of the orchestra.* *’* (Pp5)

The benefit of distributed leadership and a facilitative, servant leader at the helm of a less hierarchical PCN was recognised by the participants:

*‘* *Absolutely, systematic, servant leadership, yeah we need all that.* *’* (Pp9)

*‘* *...* *for the leaders it’s much more facilitative and supportive and that’s still bit hierarchical in that they’re always going to turn to someone and say* *‘* *help* *’* *and how do we do that* *—* *in a more facilitative way, but there’s little outside of the clinical work that needs that hierarchical approach.* *’* (Pp7)

Although not defined as such, the challenge of ‘hybrid’ managers was raised, with a concern that the public may perceive PCN leaders as being taken away from vital clinical work:

*‘* *If we are creating clinical leaders, then we are taking clinicians away from patients, and then that generates more pressure in the system. Now I personally don’t think that’s a bad thing, I’ve seen really good examples of combining clinical and non-clinical leadership causing accelerations of improvements* *…* *for many it feels counterintuitive and you’ve got to be able to rationalise that and explain that and reassure people that it’s OK.* *’* (Pp8)

## Discussion

### Summary

The findings suggest that PCN leaders appear to be walking a tightrope between a traditional hierarchical response to the challenges of contracting, workforce, and variation in quality across general practice, and a networked response to meeting needs, collaborating for acuity, and innovating with local assets. The responders highlighted the sensitivities of balancing creativity versus a top-down ‘command-and-control’ mandate. However, there was an overwhelming support voiced for PCNs remaining flexible and adaptive, and retaining local control in the face of central NHSE directives, in order to best serve the needs of its population.

### Strengths and limitations

Although the aim of qualitative research is not to argue generalisability, given that the goal of PCNs is to bring a range of clinical professional groupings together, the transferability of the findings may be limited as only GPs were interviewed. The sample was self-selected in that the participants played a significant role in the leadership and organisation of their PCN and therefore arguably displayed greater engagement with the reorganisation initiative. The sample of participants is small, but nonetheless the in-depth interviews captured reflections across nine PCNs, with further insights generated by survey response triangulation. Future research to ascertain quantifiable impact of PCNs on the quality of clinical care and disease management is needed and PCNs are in the process of developing clear areas of work, including needs-based definitions, for this purpose.

### Comparison with existing literature

Parallels can be drawn here on pre-existing literature on the benefits of retaining flexibility in networked organisations in public sector provision, for instance, to allow for greater responsiveness to demand.^21^ In order to become a successful network, the participants’ greatest identified requirement was for time and support to establish the relationships they needed to make effective collective decisions. The need for time and tensions stemming from clashing temporal demands have already been highlighted in the literature.^22^ Therefore, the findings suggest challenge to policy assumptions around the ease and speed with which new collaborations are formed. Indeed, it has been well documented that time is needed for trust-building, effective relational working, and to overcome siloed activity;^23^ all highlighted by the participants as key factors in establishing new organisational forms.

Further, the PCN leaders identified the need to move upstream to prevention, as well as multidisciplinary approaches to people slipping through the net with complex needs. This would be greatly enabled by access to data to understand local need to enable PCNs to move from reactive to proactive activity. The Marmot Review 10 years on identifies the urgency of this work.^24^ The present study’s findings also reflect tensions at the time of the inception of PCNs, whereby ‘top-down’ heavily task-oriented NHSE mandates were seen by some as being at odds with the reality of general practice, as demonstrated by pressure from GP leaders to scrap initial PCN draft-service specifications owing to their ‘impossible’ targets.

It can be argued that that question at the heart of this is whether PCNs as new organisational forms are delivery mechanisms or real collaborative networks. Malby and Anderson-Wallace^6^ identified that the PCN leadership challenge lies in the distinctiveness of networks, which is in part reliant on the distribution of power and leadership across members. No doubt as expressed by the participants, this requires a collaborative, facilitative, and democratic style with the PCN leads being confident in dealing with conflict, and able to make the most of diverse members’ views and assets. As the pandemic hit, the NHS acted quickly to *’* *…* *expand capacity and reorganise services to help ensure that it can cope* *’*.^25^ The need for interagency liaisons with multidisciplinary teams, patients, and carers at the local level has become even more crucial.

Moreover, as a more collaborative model of organising in the NHS is moved into, the findings hold further implications for clinical leadership. Whether this has been accelerated by the change in policy direction from competition to collaboration is unknown. However, as echoed by the participants, there is a climate for collaboration that embraces both a more collegiate relationship with general management and across the clinical professions (and now including social care as part of the ICS agenda). This is particularly seen in the emerging GP leadership in CCGs where GPs appear to be more likely to adopt collaborative and facilitative over ‘heroic’ leadership styles.^26^ Leadership in high-performing health systems is more likely to be distributed, and therefore in focusing on clinical leadership development for the future the model of development should not just be for senior leaders, but for the full range of clinical leaders working at multiple levels and in multidisciplinary teams contributing to securing quality health care for all. This is supported by West and West’s^27^ report on leadership in the NHS, which states that successful organisations are ‘leader-ful’ not just ‘well-led’. Therefore, it follows that in addressing clinical leadership effectiveness, organisations need to provide clinical leadership in an integrated multidisciplinary model across all levels of organisational decision making.

### Implications for practice

While the mandate from the NHS is ‘efficiencies’ of scale, participating PCN leaders reported focusing on needs and the complexity of the work that can only be delivered at scale. This is a subtle but important difference, with the former alluding to health service efficiencies and the latter to providing quality services. Overall, this study found that there were clear, although still emergent, areas of focus for PCNs and their leaders as they navigated these new organisational forms, namely to meet complex population needs through integration; to secure access for marginalised people; to improve the quality of care for the whole population; to reduce unwarranted variations; and to support sustainable general practice and a resilient workforce. In the pandemic context, PCNs have been praised for their concerted vaccination centre coordination, addressing the population health agenda with locally sensitive responses and visible leadership.^28^ While the desire to be proactive and collaborative has been emphasised, the importance of distributed leadership and time given to building trust and effective working relationships within new organisational forms cannot be underestimated.
